# Welcome to CIRSE’s new journal for endovascular therapies

**DOI:** 10.1186/s42155-018-0014-4

**Published:** 2018-06-28

**Authors:** Jim A. Reekers

**Affiliations:** Department of Interventional Radiology, Academic Medical Center, Amsterdam, Netherlands

Over the past 40 years, Interventional Radiology (IR) has been the driving force for innovation and the continuous invention of new procedures. Ever since Charles Dotter defied sceptics and insisted that angiography could be used to actively treat patients, the revolutionary concept of “catheter therapy” sparked the development of a new area of medicine which was based on a minimally invasive image guided approach, finding its way slowly but surely into clinical practice.

Today, many of the endovascular procedures invented by IR have entered routine clinical practice and are being continually improved. For the Interventional Radiologist who does not use a scalpel, innovation and refinement of endovascular techniques has always been, and continues to be, the only way forward. It is this spirit which makes IR so unique.

I still remember the early days, with CIRSE meetings of less than 300 delegates in one lecture room, where new developments were discussed and the conversations continued outside the congress centre, in hotel bars, around swimming pools and during dinners. The flow of innovative ideas never stopped. In 1978, CVIR was established and provided ample room for case reports and small case series. Those were exciting and inspiring times. We were friends belonging to the same club, passionate about what we did and how Interventional Radiology was about to change the way patients can be treated.

Today the CIRSE Annual Congress welcomes over 6500 delegates and Interventional Radiology has become an important discipline which cannot be ignored in any modern hospital. CIRSE’s official journal CVIR has become a very important, high impact journal covering the entire spectrum of Interventional Radiology. I can say that it really was a pleasure to have been actively involved in all these great developments.

However, with the increase in abstracts received for the CIRSE congresses and the number of manuscripts submitted to CVIR, the chance to be accepted for presentation and publication has decreased. At the same time, the regulatory environment has changed and the call for evidence-based science has gotten louder. The podium for new ideas, small case series, first in man, hypothesis, short communications and study protocols, has become very small.

CIRSE however, has recognized and understood how essential communication is for the continuous and stable development of IR and Endovascular Therapies. Our new journal, CVIR Endovascular will therefore offer a new low-entry podium for communications with your peers and is intended for all specialists working in the field of endovascular treatment.

CVIR Endovascular is an open access journal, in-line with how IR’s have always communicated, openly sharing new information with everybody. The open access model of the journal will offer a fast and reliable way to disseminate knowledge, allowing you to reach readers around the world. It also means that your paper will be freely available for everyone immediately upon publication.

Furthermore, CVIR Endovascular chose to introduce a new peer-review model. To encourage discussions, the journal has decided to have reviewer comments published together with the article and displayed for everyone to read and comment on. We believe that this will change the reviewer’s role from a “quick judge” to a mentor, allowing authors and readers alike to access useful additional information.

It is important to offer this new publication opportunity within the CIRSE journal family. We have no page limitations and can reach out to the all medical specialists in the field of endovascular treatment.

I therefore cordially invite all of you to use this new publication and to submit your mansucripts and case reports to CVIR Endovascular and thus help us to make this journal successfull as a forum where all endovascular groups come together.

